# A Novel Virtual Reality-Based Training Protocol for the Enhancement of the “Mental Frame Syncing” in Individuals with Alzheimer's Disease: A Development-of-Concept Trial

**DOI:** 10.3389/fnagi.2017.00240

**Published:** 2017-07-27

**Authors:** Silvia Serino, Elisa Pedroli, Cosimo Tuena, Gianluca De Leo, Marco Stramba-Badiale, Karine Goulene, Noemi G. Mariotti, Giuseppe Riva

**Affiliations:** ^1^Department of Psychology, Università Cattolica del Sacro Cuore Milan, Italy; ^2^IRCCS Istituto Auxologico Italiano, Applied Technology for Neuro-Psychology Lab Milan, Italy; ^3^Department of Clinical and Digital Health Sciences, College of Allied Health Sciences, Augusta University Augusta, GA, United States; ^4^Department of Geriatrics and Cardiovascular Medicine, IRCCS Istituto Auxologico Italiano Milan, Italy

**Keywords:** allocentric, egocentric, spatial memory, Alzheimer's Disease, virtual reality

## Abstract

A growing body of evidence suggests that people with Alzheimer's Disease (AD) show compromised spatial abilities. In addition, there exists from the earliest stages of AD a specific impairment in “mental frame syncing,” which is the ability to synchronize an allocentric viewpoint-independent representation (including object-to-object information) with an egocentric one by computing the bearing of each relevant “object” in the environment in relation to the stored heading in space (i.e., information about our viewpoint contained in the allocentric viewpoint-dependent representation). The main objective of this development-of-concept trial was to evaluate the efficacy of a novel VR-based training protocol focused on the enhancement of the “mental frame syncing” of the different spatial representations in subjects with AD. We recruited 20 individuals with AD who were randomly assigned to either “VR-based training” or “Control Group.” Moreover, eight cognitively healthy elderly individuals were recruited to participate in the VR-based training in order to have a different comparison group. Based on a neuropsychological assessment, our results indicated a significant improvement in long-term spatial memory after the VR-based training for patients with AD; this means that transference of improvements from the VR-based training to more general aspects of spatial cognition was observed. Interestingly, there was also a significant effect of VR-based training on executive functioning for cognitively healthy elderly individuals. In sum, VR could be considered as an advanced embodied tool suitable for treating spatial recall impairments.

## Introduction

It is traditionally accepted that humans are able to represent space and recall spatial information with two fundamental spatial representations: one comprising information about the position of the individual in relation to the surrounding objects (i.e., egocentric), and the other one including information about the position of the objects relative to each other in the environment (i.e., allocentric). For example, to recall the spatial position of a supermarket, individuals may simply refer to turning “left” or “right” since these directions strictly depend first of all on the spatial position of their own body (i.e., egocentric reference frame). However, since the position of an object “changes” according to the spatial position of our own body in the space, individuals, in order to refer to the supermarket, also need to have a stored spatial layout that includes the relationships between two external landmarks (i.e., allocentric reference frame). A neurocognitive model was recently advanced to explain how spatial recall works (Burgess et al., 2001; Byrne et al., 2007). Burgess and his colleagues explained that, in the presence of a spatial cue, allocentric representation is retrieved through a process of pattern completion. Though initially allocentric, this representation is translated to egocentric for navigation purposes in the medial parietal areas via information provided by other cells (Hartley et al., 2014). More specifically, the retrosplenial cortex (RSC) is responsible for the transformation of long-term hippocampal allocentric representations into egocentric parietal representations to account for the rotational offset between the different spatial coordinates (Maguire, 2001; Vann et al., 2009). While parahippocampal regions are involved in processing the visuo-spatial structure of the spatial scene, RSC supports the process of spatial recall thanks to the retrieval of reference that allows the scene to be localized within the wider spatial environment (Epstein et al., 2007; Ekstrom et al., 2014).

Studies on spatial recall provide a unique opportunity to better understand topographical disorientation that may occur in both physiological and pathological aging (Moffat, 2009; Gazova et al., 2012), especially in Alzheimer's Disease (AD; Lithfous et al., 2013; Serino et al., 2014b). In patients with AD, there is a main impairment in episodic memory functioning due to neurodegeneration in medial temporal structures (e.g., entorhinal cortex, hippocampal formation, parahippocampal gyrus; for review, see Tromp et al., 2015). This episodic memory disorder is temporally and spatially related to both the distribution of neurofibrillary tangles within the medial temporal lobe and the volumetric loss of the hippocampus (Braak and Braak, 1995; Dubois et al., 2007). However, due to these neurodegenerative processes, episodes of topographical disorientation are also common since they are related to an early deficit in allocentric recall both in patients with amnestic mild cognitive impairment (aMCI) and with AD (for review, see Serino et al., 2014b). In addition, there exists from the earliest stages of AD the presence of a specific impairment in the ability to encode and store an allocentric hippocampal representation and then to translate it into an egocentric parietal representation. Serino and Riva (2013, 2014) proposed that there is a specific cognitive process (i.e., the “mental frame syncing”) underlying this egocentric-allocentric transformation that may be useful in supporting the recall of spatial scenarios. The mental frame syncing may be conceived as a cognitive process that permits the transformation from a stored allocentric viewpoint-independent representation (including the above mentioned object-to-object information) to an egocentric one by computing the bearing of each relevant “object” in the environment in relation to the stored heading in space (i.e., information about our viewpoint contained in the viewpoint-dependent representation). Serino and Riva (2013, 2014) argued that patients with AD might experience a break in the “mental frame syncing” due to neurodegenerative processes that affect the medial temporal lobe and the hippocampus in particular. Indeed, neurofibrillary tangle deposition leads to degeneration of hippocampal subfields, first the CA1 and then the subiculum, CA2, CA3, and CA4 (Bartsch and Wulff, 2015). Padurariu et al. (2012) found neuronal loss especially in the CA1 and CA3, which elaborates allocentric representations containing pure object-to-object information of the spatial scene and those containing information about the individual's viewpoint within the spatial scene, respectively (Behrendt, 2013).

In this context, Virtual Reality (VR) could be considered a new advanced tool for specifically assessing and treating spatial recall impairment (Burgess et al., 2002; Bohil et al., 2011; García-Betances et al., 2015; Serino et al., 2015b).

A virtual town center where subjects can wander around, find an object and memorize its spatial position using an HMD and a gamepad is an example of how a VR environment can be used to assess and treat spatial recall deficiencies among subjects with AD. Indeed, in virtual environments it is possible to easily implement a “reorientation task,” which is characterized by two phases. In the *encoding phase*, participants are instructed to memorize the position of an object. Then, in the *retrieval phase*, participants have to indicate the position of the object, starting from another position. As suggested by Bosco et al. (2008), this strategy induced interference in the egocentric representation of the object with respect to the participants' view (i.e., “virtual disorientation”). To indicate the position of the object, this technique forced the participants to refer to their allocentric viewpoint-independent representation and sync it with the allocentric viewpoint-dependent representation. In a recent study, Serino et al. (2015b) used a virtual task based on this paradigm and found that patients with AD have specific impairment in the synchronization of allocentric viewpoint-independent and viewpoint-dependent representation. In this study, participants were asked to memorize the position of an object after having entered a virtual room. Then they were asked to recall its position in two different tasks. The first task involved an aerial map of the room (i.e., a task that measures the ability to store an allocentric viewpoint-independent representation); the second task involved entering the empty version of the virtual room again, but this time from another starting point (i.e., a task that measures the ability to sync the stored allocentric viewpoint-independent representation with the viewpoint-dependent one). Results revealed that while aMCI patients showed a deficit only in the first task, a more profound deficit was found for patients with AD; namely, they were not able to synchronize the two different representations.

As concerns rehabilitation, some recent systematic reviews and meta-analyses have provided support for the efficacy of cognitive training in improving the quality of life and careers of patients with AD. However, conclusions drawn from existing studies must be viewed with caution due to the limited number of randomized controlled trials and their methodological limitations (Clare and Woods, 2004; Olazarán et al., 2010; Bahar-Fuchs et al., 2013).

As reviewed by García-Betances et al. (2015), VR can also be used as a rehabilitative tool for patients with AD, but very little work has been done on this topic so far. In general terms, the first great advantage offered by VR is its possibility to develop tailored cognitive exercises within meaningful environments (Riva et al., 2006), which is particularly relevant since cognitive training might be particularly demanding for patients with AD. More specifically, in virtual environments it is possible to implement cognitive training based on specific rehabilitative mechanisms. For example, in our cognitive training we exploited the technique known as “virtual disorientation” (as described before), which is difficult to set up in a real-life situation.

An interesting example was offered by Kober et al. (2013), who found that VR can be a useful tool to implement a cognitive training program for spatial abilities when also used with passive navigation. In a study in which eleven neurologic patients with focal brain lesions and topographical disorientation performed a route finding task, results showed that a VR-based verbally guided passive navigation training program was able to improve general spatial abilities in neurologic patients. Caglio et al. (2012) showed that a virtual navigation task led to improvements in memory function in a 24-year-old man with a traumatic brain injury; this improvement corresponded to increased activity in cerebral structures crucial for both navigation and memory, such as the hippocampus and parahippocampus. However, no studies have explored the potentiality of VR for training the cognitive ability to synchronize different spatial representations in patients with AD.

Based on these premises, the main objective of this development-of-concept trial^1^(Dobkin, 2009) is to evaluate if a VR-based training protocol specifically focused on the enhancement of the “mental frame syncing” between allocentric viewpoint-dependent and viewpoint-independent representation would be able to improve general spatial abilities in a sample of patients with AD. In order to preliminarily evaluate the efficacy of the novel VR-based training, two groups of patients with AD (i.e., “VR-based training” vs. “Control Group”) will be compared using a traditional neuropsychological battery before and after both training programs. Moreover, eight cognitively healthy elderly individuals were recruited to participate in the VR-based training in order to have a different comparison group.

## Materials and methods

### Participants

We recruited 20 elderly subjects (age > 65 years old) from a social senior center located in Milan (Italy) from individuals referred as meeting the NINCDS-ARDRA criteria (McKhann et al., 1984). They were evaluated with the Milan Overall Dementia Scale (Brazzelli et al., 1994) and only individuals who had a score under 85.5 (i.e., the clinical cut-off for probable dementia) were included in the study. Participants were randomly assigned to “VR-based training” (“VR Group-AD”; *n* = 10) or “Control Group” (“Control Group-AD”; *n* = 10). We also recruited eight cognitively healthy elderly subjects (age > 65 years old) from the same setting (i.e., a social senior center located in Milan, Italy) to participate in the VR-based training (“VR Group-Normal Aging”; *n* = 8). They were evaluated with the Milan Overall Dementia Scale (Brazzelli et al., 1994) and only individuals who had a score over 85.5 were included in the study.

Exclusion criteria for the three groups were: (1) presence of visual and balance deficits which may interfere with the use of VR technology; and (2) the additional presence of psychiatric disorders or other neurological conditions, such as traumatic brain injuries or strokes.

The VR Group-AD included nine women and one man, while the Control Group-AD included eight women and two men (χ^2^ = 0.392; *p* = 0.531). The mean age for the VR Group-AD was 86.60 (*SD* = 6.13), with mean years of education of 9.80 (*SD* = 3.97); the mean age for the Control Group-AD was 88.70 (*SD* = 3.59), with mean years of education of 7.00 (*SD* = 5.00). There were no significant differences between two groups in terms of age [*t*_(18)_ = 1.675; *p* = 0.111] or education [*t*_(18)_ = −0.934; *p* = 0.362]. The VR Group-Normal Aging comprised four women and four men. The mean age for this group was 86.62 (*SD* = 6.19), with mean years of education of 9.12 (*SD* = 5.05). There were no significant differences between the VR Group-AD and VR Group-Normal Aging in terms of age [*t*_(16)_ = 0.318; *p* = 0.755] or education [*t*_(16)_ = −0.009; *p* = 0.993]. All participants provided written informed consent, which was approved by the Ethical Committee of IRCCS Istituto Auxologico Italiano. The study was conducted in compliance with the Helsinki Declaration of 1975, as revised in 2008.

### VR-based training program

**Figure 1:**
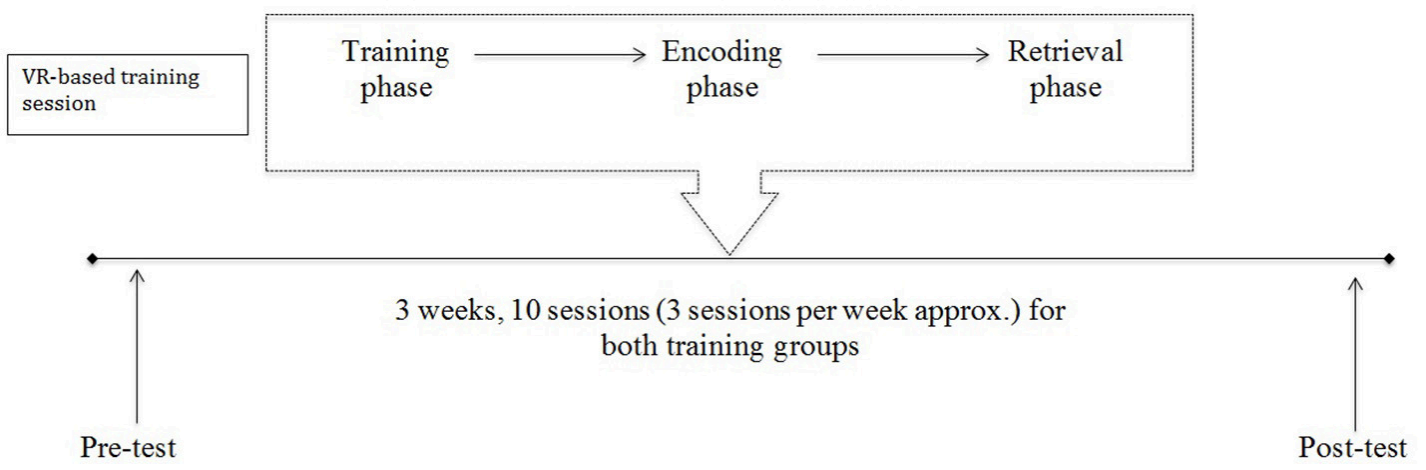
Timeline of the VR-based training program. The training consisted of 10 sessions for 3–4 consecutive weeks, with approximately three sessions a week. After brief training in VR technology (about 2 min), each session comprised two parts: an “*encoding*” and a “*retrieval phase*.” Participants were assessed with a comprehensive neuropsychological assessment before and after participation in the training.

A Virtual Reality (VR)-based protocol was developed to train the ability in syncing between allocentric viewpoint-dependent and allocentric viewpoint-independent representations (Serino and Riva, 2013, 2014). The training program consisted of 10 sessions for 3–4 consecutive weeks, with approximately three sessions a week. Each session contained an “encoding phase” and a “retrieval phase” (see Supplementary Material). The first and last sessions were also devoted to the administration of a neuropsychological assessment (see Figure 1).

**Figure 2:**
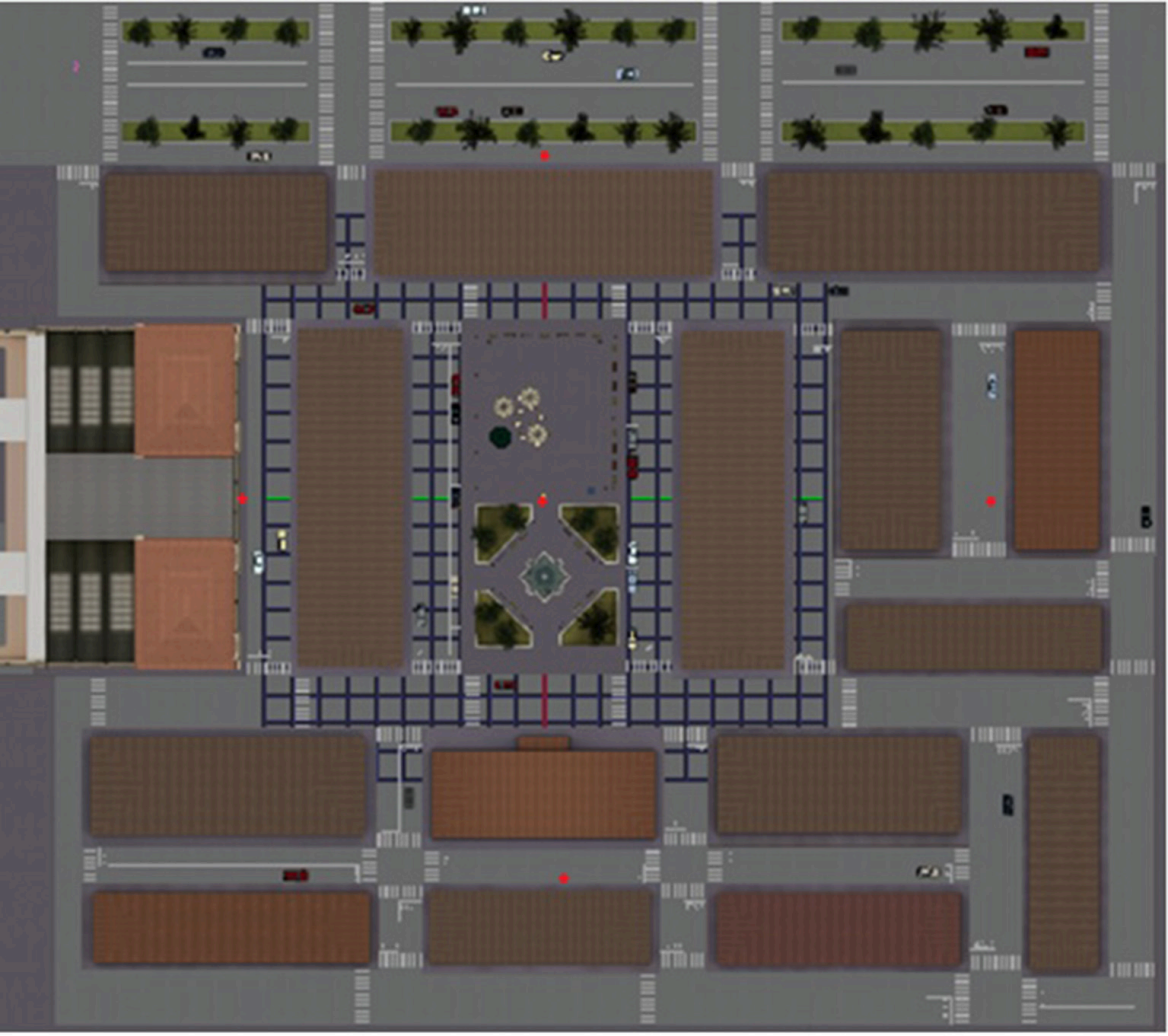
The map of the virtual city. The city was built around a central square with a fountain and a bar with some tables, which represents the starting point of the navigation. There were buildings and shops spread out in the city. In the northern part of the city, there was a large street surrounded by trees with some cars, whereas the southern part was more residential.

**Figure 3:**
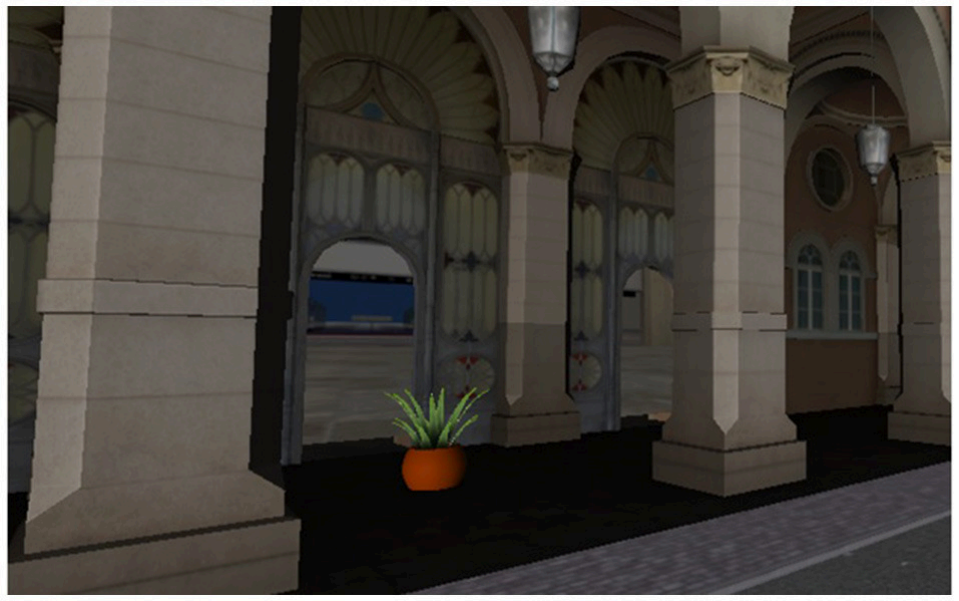
One of the objects to be found during the VR-based training program. During the encoding phase, participants were asked to locate one, two or three hidden objects (i.e., a bottle of milk, a plant in a vase and a trunk). They received the specific instruction of memorizing the position of these objects, which were positioned at different parts of the city since in the retrieval phase they were asked to retrieve their spatial positions. The training was personalized according to the level reached by each participant, so that if a patient was not able to locate the first object, the other objects were not presented.

Each VR-based treatment session had the same structure. All the sessions were carried out in a quiet room in the social senior center; participants were invited to enter and take a seat in front of a table where the PC was positioned (see the Section Procedure for other technical details). They were then asked to take a gamepad and to navigate inside the virtual environment, thus starting the session. After initial training in VR technology (i.e., a different virtual city where participants were assisted in learning how to navigate within virtual environments using the gamepad), the encoding phase started. A virtual city had been developed as the rehabilitation environment. It was built around a central square with a fountain and a bar with some tables, which represents the starting point of the navigation (see Figure 2). There were buildings and shops spread out in the city; no human characters were present in order to avoid possible interference in the rehabilitation process. The neuropsychologist asked participants to enter this virtual city starting from its center to discover one, two or three hidden objects (i.e., a bottle of milk, a plant in a vase and a trunk; see Figure 3). Participants were specifically instructed to memorize the positions of these objects, which were placed at different parts of the city (see Table 1).

**Table 1:** The objects with their spatial location in the virtual city in the encoding phase and the starting point of the participants, after the virtual disorientation, in the retrieval phase.

	**Session 1**	**Session 2**	**Session 3**	**Session 4**	**Session 5**	**Session 6**	**Session 7**	**Session 8**	**Session 9**	**Session 10**
**ENCODING PHASE**										
Objects' Position	West	West; East	West; East	West; East	South; North; East	West; East	West; East	West; East	North; South; West	South; East; West
**RETRIEVAL PHASE**										
Starting point	East	North	South	East	West	South	North	South	East	Center

*After the fifth session, the number of possible objects to be memorized remains fixed to two for three sessions (which means that there was not an increase of difficulty) because of the absence of the facilitation offered by the interactive aerial view during the retrieval phase*.

The number of the objects to be memorized depended on the level reached by each participant; if the patient was not able to locate the first object, the other objects were not presented. There was no time limit in the encoding phase, but all participants found the object(s) in ~10–15 min. Next, in the retrieval phase, they were asked to retrieve the position of the objects identified in the first phase (which were now absent) once they entered the virtual city from another starting point. There was no time limit in the retrieval phase. Also in this case, all participants retrieved the position of the object(s) in ~10–15 min; they were instructed to stop and tell the neuropsychologist when they thought they had reached the correct position. If participants were not able to retrieve the correct position of the object, the neuropsychologist helped them by guiding them in the virtual environment in order to strengthen the amnestic trace.

The entire VR-based session treatment lasted about 20 min. For each session, the neuropsychologist filled in a grid indicating which object(s) was/were found and briefly describing the progress reached during the session. It was essential to record the number of objects to be included in each session because, as previously explained, if patients were not able to find the first object, the other objects were not presented. Because patient response to VR-based training can vary greatly, cognitive exercises should be personalized according to a patient's specific needs.

**Figure 4:**
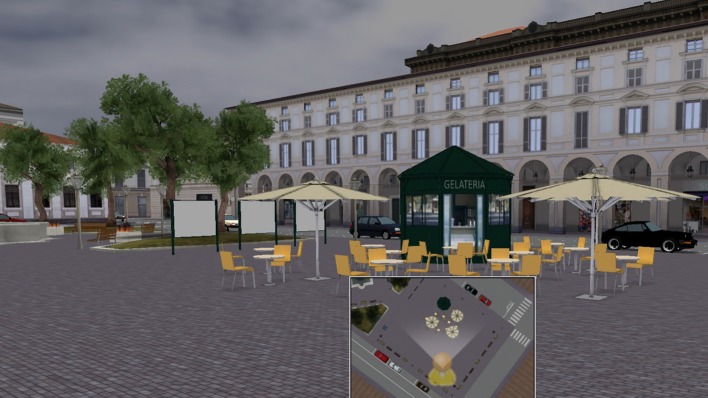
During the retrieval phase, to facilitate the retrieval of spatial information, an interactive aerial view of the virtual city was presented during the navigation and the display was oriented depending on the participant's movement in the virtual world.

In the first five VR treatment sessions, an interactive aerial view of the virtual city was displayed during the retrieval phase; its display was oriented depending on the participant's movement. The aerial view of the city was on the left part of the screen of the PC, and the participants could see in every direction for a distance of 25 m, which was the maximum distance at which any object could be seen (see Figure 4).

Moreover, when needed, the neuropsychologist helped patients with the gamepad used to navigate in the virtual city or provided little clues in order to avoid errors. Indeed, de Werd et al. (2013) underlined the importance of “errorless learning” in people with AD and severe memory impairments.

This VR-based training was developed using the open-source based software NeuroVirtual 3D, a recent extension of the software NeuroVR (Riva et al., 2011; Cipresso et al., 2014). The software (http://www.neurovr3.org/) is composed of two main modules: the Editor, which permits the customization of pre-designed virtual environments (e.g., a city, an apartment, a supermarket, etc.) to the specific needs of an experimental setting; and the Player, which allows the administration of the configured virtual environments. Thanks to the Editor, researchers can customize virtual environments by choosing the appropriate stimuli from a database of objects (both 2D and 3D objects, videos, and sounds). No programming skills are necessary since there is a user-friendly icon-based interface that allows researchers to easily put the stimuli in the pre-designed virtual environments. When ready, the configured virtual environments can be visualized via the Player both in immersive or not-immersive modalities.

### Outcome measures

To obtain detailed information across a wide range of cognitive domains before and after both training programs, all participants underwent a detailed neuropsychological assessment conducted by an experienced neuropsychologist. These measures included (1) the phonemic verbal fluency and categorical verbal fluency test (Novelli et al., 1986) and Frontal Assessment Battery (FAB; Appollonio et al., 2005) for the evaluation of executive functions; (2) the Attentional Matrices Test (Spinnler and Tognoni, 1987) for measuring selective attention; (3) the Digit Span Test (Monaco et al., 2012) for assessing short-term memory abilities; (4) and the Corsi Block Test (Corsi, 1973; Monaco et al., 2012) in both its versions (i.e., Corsi Span and Corsi Supraspan) for the assessment of short and long-term spatial memory abilities. All scores obtained from the neuropsychological battery were corrected for age and education level according to Italian normative data.

### Procedure

After participants gave their informed consent to be included in the study, one group of patients with AD was randomly assigned to the “VR-based training” (“VR Group-AD”), while the other group of patients with AD was randomly assigned to the “Control Group-AD” (“Control Group-AD”). Control Group-AD underwent the traditional cognitive rehabilitative activities with the neuropsychological staff at the social senior center (i.e., cognitive stimulation programs, such as cards games, naming, fluency, and music listening). A group of cognitively healthy individuals were recruited to participate in the VR-based training (“VR Group-Normal Aging”).

All participants were then given the Mini-Mental State Examination (MMSE; Folstein et al., 1983) and the neuropsychological battery in order to obtain an overview of their general cognitive functioning. As specifically regards the VR group, participants were asked to sit in front of a portable computer (ACER ASPIRE with CPU Intel® Core™i5 and graphic processor Nvidia GeForce GT 540M, 1024 × 768 resolution). The participants were also given a gamepad (Logitech Rumble F510) that allowed them to explore and interact with the environment. After the training phase in VR technology (~2 min), the VR-based treatment session started. Both training programs consisted of 10 sessions for 3–4 consecutive weeks, with approximately three sessions a week. At the end of both training programs, the same neuropsychological assessment was administered to all participants.

### Data analysis

To evaluate the efficacy of the novel VR-based training, we first compared the performances on the neuropsychological assessment between the two groups of patients with AD. To ensure that there were not differences in performance on the neuropsychological tests before the participants underwent the training programs, a series of Mann–Whitney *U-*tests were carried out on participants in the “VR Group-AD” and “Control Group-AD” on these variables. Then we computed the differences between pre- and post-test scores on the neuropsychological assessment (delta scores). Then, a Mann–Whitney *U-*test between the “VR group-AD” and “Control Group-AD” on each computed delta score was carried out.

Moreover, we compared the performances on the neuropsychological assessment between the patients with AD who participated in the VR-based training program with those of cognitively healthy elderly individuals. After the comparison between the two groups on the neuropsychological assessment before participating in VR-based training, a Mann–Whitney *U-*test between “VR group-AD” and “VR Group-Normal Aging” on computed delta scores for each neuropsychological test was carried out.

Given the small sample sizes, comparisons between groups were most suited to non-parametric testing for all our analyses (Siegel and Castellan, 1988). All statistical analyses were performed using the Statistical Package for the Social Sciences for Windows (SPSS Inc., Chicago, IL, USA), version 23. A *p* < 0.05 was considered statistically significant.

## Results

### Comparison between the two groups of patients with AD—baseline characteristics

Participants' scores obtained from the neuropsychological assessment and the statistical comparisons are shown in Table 2.

**Table 2:** Baseline characteristics.

	**VR group-AD**	**Control group-AD**	***z*^c^**	***p*^d^**	***r*^e^**
MMSE^a^	22.05 (1.62)	20.79 (1.80)	−1.592	0.111	0.355
Verbal fluency test	19.90 (4.86)	15.90 (5.84)	−1.290	0.197	0.288
Verbal categorical test	23.20 (3.22)	19.90 (5.90)	−1.367	0.172	0.306
FAB^b^	11.18 (2.75)	10.36 (3.34)	−0.832	0.406	0.186
Attentional matrices test	20.80 (8.19)	18.38 (3.90)	−0.756	0.406	0.169
Digit span test	6.30 (1.51)	5.57 (0.57)	−0.683	0.495	0.153
Corsi block test—span	3.91 (1.02)	4.08 (0.57)	−0.456	0.648	0.102
Corsi block test—supraspan	6.83 (1.71)	7.97 (1.89)	−0.756	0.450	0.169

*Scores obtained from the first neuropsychological assessment for patients with AD assigned to the VR-based training (“VR Group- AD”) and for patients with AD assigned to the “Control Group-AD” (“Control Group-AD”). Data are shown as means and standard deviations (SD)*.

a *MMSE, Mini Mental State Examination*.

b *FAB, Frontal Assessment Battery*.

c *Mann–Whitney U testing*.

d *p-value*.

e *effect size (|r| > 0.40 can be considered as a medium effect size, Cohen, 1988)*.

Results from the Mann–Whitney *U-*test indicated no significant differences between the two groups of patients with AD on the neuropsychological tests administered before the beginning of both training programs. Accordingly, the VR Group-AD and Control Group-AD were comparable as concerns the baseline characteristics.

### Comparison between the two group of patients with AD—pre- and post-neuropsychological assessment

To investigate the efficacy of the novel VR-based training, the differences between pre- and post-test scores on the neuropsychological assessment (delta scores) between the two groups were evaluated using the Mann–Whitney *U-*Test.

As shown in Table 3, VR Group-AD scores were significantly better than Control Group-AD scores on the Corsi Block Test—Supraspan. Regarding all of the other neuropsychological measures administered, VR group-AD performance did not differ significantly from that of Control Group-AD. Although not significant, a great improvement for VR Group-AD was noticeable in attentional abilities, as underlined by the higher delta score on the Attentional Matrices Test.

**Table 3:** Pre-post assessment.

	**VR group-AD**	**Control group-AD**	***z*^b^**	***p*^c^**	***r*^*d*^**
Verbal fluency test	−1.60 (4.00)	0.20 (4.21)	−0.836	0.403	0.187
Verbal categorical test	0.70 (4.71)	0.50 (4.21)	−0.038	0.970	0.008
FAB^a^	0.84 (4.26)	−0.66 (1.94)	−0.725	0.468	0.162
Attentional matrices test	4.71 (9.52)	0.70 (2.89)	−1.067	0.286	0.239
Digit span test	0.01 (0.82)	−0.33 (0.90)	−0.728	0.466	0.162
Corsi block test—span	0.17 (1.20)	0.14 (1.21)	−0.084	0.933	0.019
Corsi block test—supraspan	1.56 (2.53)	−0.01 (1.43)	−2.120	0.035	0.474

*Delta scores obtained from the pre-post neuropsychological assessment for patients with AD assigned to the VR-based training (“VR Group-AD”) and for patients with AD assigned to the “Control Group-AD” (“Control Group-AD”). Data are shown as means and standard deviations (SD)*.

a *MMSE, Mini Mental State Examination*.

b *Mann-Whitney U testing*.

c *p-value*.

d *effect size (|r| > 0.40 can be considered as a medium effect size, Cohen, 1988)*.

### Comparison between patients with AD and cognitively healthy controls assigned to VR-based training- baseline characteristics

Cognitive abilities evaluated with the neuropsychological assessment before participation in the VR-based training and the statistical comparisons between the two groups are shown in Table 4.

**Table 4:** Baseline characteristics.

	**VR group-AD**	**VR group-normal aging**	***z*^c^**	***p*^d^**	***r*^e^**
MMSE^a^	22.05 (1.62)	27.73 (2.02)	−3.560	<0.001	0.839
Verbal fluency test	19.90 (4.86)	22.87 (10.16)	−0.801	0.423	0.189
Verbal categorical test	23.20 (3.22)	25.75 (9.42)	−0.490	0.624	0.155
FAB^b^	11.18 (2.75)	13.12 (3.34)	−1.422	0.155	0.335
Attentional matrices test	20.80 (8.19)	29.42 (10.11)	−1.599	0.110	0.379
Digit span test	6.30 (1.51)	5.63 (0.55)	−0.489	0.625	0.115
Corsi block test—span	3.91 (1.02)	4.62 (0.60)	−1.780	0.075	0.419
Corsi block test—supraspan	6.83 (1.71)	9.25 (1.89)	−2.577	0.010	0.607

*Scores obtained from the first neuropsychological assessment for patients with AD assigned to the VR-based training (“VR group- AD”) and for cognitively healthy elderly individuals assigned to the VR-based training (“VR Group-Normal Aging”). Data are shown as means and standard deviations (SD)*.

a *MMSE, Mini Mental State Examination*.

b *FAB, Frontal Assessment Battery*.

c *Mann–Whitney U testing*.

d *p-value*.

e *Effect size (|r| > 0.40 can be considered as a medium effect size, Cohen, 1988)*.

Results from the Mann–Whitney *U-*test showed that patients with AD manifested greater difficulties in general cognitive functioning and in long-term spatial memory, as measured by MMSE and Corsi Block Test—Supraspan, respectively. It is important to note that although there were not significant differences between the two groups in the other cognitive domains, the mean scores of the cognitively healthy elderly individuals appeared above the clinical cut-off (Verbal fluency test: 17; Verbal categorical test: 25; Corsi Block Test—Span: 3.75; Digit: 3.75) or near the clinical cut-off (FAB: 13.50 and Attentional Matrices: 30).

### Comparison between patients with AD and cognitively healthy controls assigned to VR-based training—pre- and post-neuropsychological assessment

Differences between pre- and post-test scores on all neuropsychological tests (delta scores) were evaluated using the Mann–Whitney *U-*Test (Table 5).

**Table 5:** Pre-post assessment.

	**VR group-AD**	**VR group-normal aging**	***z*^b^**	***p*^c^**	***r*^d^**
Verbal fluency test	−1.60 (4.00)	3.50 (3.89)	−2.240	0.025	0.527
Verbal categorical test	0.70 (4.71)	3.87 (5.03)	−1.206	0.228	0.284
FAB^a^	0.84 (4.26)	1.49 (2.13)	−0.670	0.503	0.157
Attentional matrices test	4.71 (9.52)	1.25 (8.53)	−0.357	0.721	0.084
Digit span test	0.01 (0.82)	−0.12 (0.83)	−0.369	0.466	0.086
Corsi block test—span	0.17 (1.20)	−0.12 (0.84)	−0.272	0.712	0.064
Corsi block test—supraspan	1.56 (2.53)	1.88 (4.21)	−0.267	0.790	0.059

*Delta scores obtained from the pre-post neuropsychological for patients with AD assigned to the VR-based training (“VR group-AD”) and for cognitively healthy elderly individuals-assigned to the VR-based training (“VR Group-Normal Aging Data are shown as means and standard deviations (SD)*.

a *MMSE, Mini Mental State Examination*.

b *Mann–Whitney U testing*.

c *p-value*.

d *Effect size (|r| > 0.40 can be considered as a medium effect size, Cohen, 1988)*.

Findings indicated a significant improvement in Verbal Fluency Test scores for cognitively healthy elderly individuals after the VR-based training. As concerns the other cognitive domains measured, although it is possible to note a general increase in the other tests evaluating executive functioning (i.e., Verbal Categorical Test and FAB), there were no other significant differences.

## Discussion

In this study, the efficacy of a novel VR-based training protocol to train the ability to sync allocentric viewpoint-dependent and viewpoint-independent representation in a sample of patients with Alzheimer's disease (AD) was evaluated. A group of cognitively healthy elderly individuals also participated in the VR-based training in order to have a different comparison group. Our results indicated a clear improvement in long-term spatial memory (as measured by the Corsi Block Test—Supraspan) after VR-based training for patients with AD; this means that transference of improvements from the VR-based training to more general aspects of spatial cognition was observed. Interestingly, VR-based training also had a significant effect on executive functioning (as measured by the Verbal Fluency Test) in cognitively healthy elderly individuals.

The first possible interpretation of our findings is that the improvement in long-term spatial memory for patients with AD may have resulted from the information provided by the interactive aerial view of the virtual city during the first phase of the training. Indeed, a recent study has found that the recall of spatial information is facilitated by the presence of an interactive aerial view of the experienced virtual environments because it provides a real-time allocentric viewpoint-dependent representation that helps individuals to place the current egocentric heading in space to compute the bearing of each relevant stored object (Serino and Riva, 2015). This “external aid” may help patients with AD improve their compromised ability to synchronize allocentric viewpoint-dependent and viewpoint-independent representation. Both active navigation and goal-directed spatial decision-making have a beneficial effect on spatial performance. In our VR-based training, participants were asked to use a gamepad to actively navigate in the virtual environments and complete a task. There was a virtual world where patients could perform actions at concrete objectives. Several studies have demonstrated the benefit of active navigation on memory performance (Brooks, 1999; Gaunet et al., 2001; Péruch and Wilson, 2004; Plancher et al., 2012). For instance, Plancher et al. (2012) asked older adults, patients with amnestic mild cognitive impairment and patients with AD to memorize a series of elements in two experimental conditions: as the driver of a virtual car (i.e., active exploration) and as the passenger of that car (i.e., passive exploration). Interestingly, for all groups, active exploration led to an enhanced recall of allocentric spatial information.

These results were generally interpreted as suggesting the role of action in enriching memory trace thanks to reinforcement of item-specific processing (Engelkamp, 1998). However, interacting with a complex environment provided participants not only with a sensorimotor trace essential for memory recall, but also with the opportunity to implement goal-directed spatial decision-making, which is another key element of active navigation. Planning spatial routes in a complex environment to achieve different objectives and, consequently, accomplish different intentions improved memory performance and appeared to be supported by the hippocampus (Viard et al., 2011).

These theoretical considerations on goal-directed spatial decision-making may offer an interesting explanation for the other findings of our work. Indeed, we also found an improvement in executive functioning of cognitively healthy elderly subjects after participation in VR-based training. By “executive function,” we refer to a complex set of cognitive abilities that includes planning, sequencing, problem-solving, and monitoring (Chan et al., 2008), strictly dependent on frontal lobe activity. According to the so-called “frontal aging hypothesis,” frontal lobes are particularly vulnerable to age-related decline (Greenwood, 2000), which leads to consequent and progressive decreases in executive functioning.

The role of frontal lobes has been demonstrated in other high-level cognitive functions, such as episodic memory (Lepage et al., 2000; Habib et al., 2003) and spatial memory (Maguire et al., 1998). In particular, as anticipated, the prefrontal cortex contribution was shown in spatial learning (Dahmani and Bohbot, 2015), and its involvement in navigation is thought to be linked to decision-making, planning and working memory (Spiers and Maguire, 2006; Moffat et al., 2007; Viard et al., 2011). For instance, Korthauer et al. (2017) found that performance on the virtual version of the Morris Water Task was associated with better verbal fluency, set switching, response inhibition, and the ability to mentally rotate objects.

It is possible that our VR-based training stimulated the two groups in different ways. In patients with AD, the training specifically stimulated the “mental frame syncing,” leading to a better spatial performance. For cognitively healthy subjects, the training enhanced frontal functioning, stimulating the implementation of goal-directed spatial decision-making to solve the proposed task.

These findings are particularly promising for AD patients because they provide support for the feasibility of VR as an effective medium for delivering spatial training programs on specific spatial skills that can be generalized to general aspects of spatial processing, which appear to become compromised early in this population. As such, VR-based interventions could be included in the wider panorama of non-pharmacological and non-invasive treatments for AD (Olazarán et al., 2010; Bahar-Fuchs et al., 2013) that promote neuroplasticity and neural reorganization through environmental enrichment (Durlach et al., 2000; Rose et al., 2005; Bohil et al., 2011; García-Betances et al., 2015; Morganti, 2015; Repetto et al., 2016). It has been demonstrated that mice exposed to environmental enrichment have shown improvements in hippocampal long-term potentiation and changes in the neuroplasticity in cerebral regions associated with learning and memory (Alwis and Rajan, 2014). More specifically, it was observed that hippocampal place cell activity was present during a virtual navigation (Harvey et al., 2009). In addition, Clemenson and Stark (2015) have recently found that 3D video games were able to improve performance on memory tasks known to be dependent on hippocampal activity.

On the other hand, our results indicated that our training also led to improvements in executive functioning, allowing the possibility to set up specific protocols for healthy elderly people aimed at improving the traditional cognitive empowerment approaches for this population (Serino and Pedroli, 2016).

Limitations must be taken into account when considering our findings. First of all, although this is a development-of-concept trial and small groups of patients are usually sufficient for this type of study, the small sample size of may limit the generalizability of our findings. Another limit of the current study is that a measure of the ability to synchronize the two described allocentric representations was not included as an outcome measure, which prevents us from investigating the relationship between neuropsychological assessments and the ability to conduct the “mental frame syncing” and from showing the progress of the patients throughout training in a graph. Indeed, only two studies introduced novel VR-based tests aimed at evaluating the presence of a deficit in “mental frame syncing,” and neither of those tests was standardized (Serino et al., 2014a, 2015b). However, it is interesting to note that preliminary results from these studies showed that general cognitive functioning (measured with MMSE) was associated with the ability to conduct the “mental frame syncing” (Serino et al., 2014a).

In sum, our results concerning the efficacy of a novel VR-based training program to improve synchronization of different spatial representations for AD are surely encouraging, but also preliminary. We have demonstrated the feasibility of our approach, but this study should be viewed as a proof-of-concept that requires further development. Summarizing researches conducted so far on patients with AD using VR technologies, García-Betances and colleagues noted that the majority of works in this field had privileged not-immersive VR-based solutions (García-Betances et al., 2015). This is not only a “technological issue,” but as future step of our approach it would be crucial to investigate its efficacy using a computer-assisted virtual environment (CAVE). In this system, virtual environments are usually projected onto the screen of a small room completely surrounding the users, thus enhancing the sense of presence (i.e., the sense of “being there;” Riva et al., 2014) and the possibility of interacting with the objects in the scene. Although there are more user-friendly, immersive and low-cost rehabilitative technologies (e.g., Oculus Rift, for a review see Castelvecchi, 2016), it is possible to develop a training program in which the interactive aerial view rotates according to the orientation of the participants' heading direction. In our previous study carried out in a CAVE, we found that the presence of a small interactive aerial view of a virtual city, including a visualized larger arrow indicating the heading direction, was able to facilitate spatial recall (Serino et al., 2015a). These outcomes emphasized the crucial role of a larger visualized arrow in order to retrieve the correct path; this arrow worked as a “external aid” in reinforcing participants' “mental frame syncing” by giving them information about their heading direction in the environment. These findings were compatible with studies elucidating the role of retrosplenial cortex in heading retrieval (Epstein, 2008; Vann et al., 2009; Marchette et al., 2014), a specific cognitive process dissociable by place recognition (Julian et al., 2015).

With these developments, controlled clinical trials involving a greater number of participants are required to provide adequate support to our proposal. Other future challenges include the inclusion of a follow-up to provide more consistent results and the elaboration of a more complex cognitive training program focusing on other cognitive skills beyond the spatial one (Clare and Woods, 2004; Bahar-Fuchs et al., 2013). Indeed, current increasing evidence on the efficacy of non-pharmacological approaches in patients with AD is sufficiently encouraging to justify additional clinical studies on this population.

## Author contributions

SS, EP, and GR developed the study concept. All authors contributed to the study design. EP, CT, and NM were involved in the data collection. SS performed the data analysis and interpretation under the supervision of GR. SS, EP, and CT wrote the first draft of the manuscript. GD, MS, KG, and GR were involved in the critical revision of the manuscript for important intellectual content. All the authors approved the final version of the manuscript for submission.

### Conflict of interest statement

The authors declare that the research was conducted in the absence of any commercial or financial relationships that could be construed as a potential conflict of interest.

## Footnotes

^1^The development-of-concept trial (which is in Phase I in the context of the Food and Drug Administration's classification of clinical trials for the pharmaceutical industry to test drugs) was aimed at preliminarily testing the efficacy of new interventions in randomized and masked pilot studies with control groups. In this kind of study, small groups of patients were usually recruited.
