# Supplementary material for: A Novel Virtual Reality-Based Training Protocol for the Enhancement of the “Mental Frame Syncing” in Individuals with Alzheimer's Disease: A Development-of-Concept Trial
Source: Front Aging Neurosci. 2017 Jul 27;9:240. doi: 10.3389/fnagi.2017.00240 (PMC5529401; doi:10.3389/fnagi.2017.00240)
Supplement: Supplementary file 2 [file Presentation1.pdf]

Supplementary Material

A novel Virtual Reality-based training protocol for the enhancement of the “mental frame syncing” in individuals with Alzheimer’s Disease: a development-of-concept trial

Silvia Serino[1,2*], Elisa Pedroli[2], Cosimo Tuena[2], Gianluca De Leo[3], Marco Stramba-Badiale[4], Karine Goulene[4], Noemi Guidone Mariotti[2,] Giuseppe Riva[1,2]

[1]Università Cattolica del Sacro Cuore, Largo Gemelli, Department of Psychology 1, 20100, Milan, Italy

[2]IRCCS Istituto Auxologico Italiano, Applied Technology for Neuro-Psychology Lab Via Magnasco, 2 20149 Milan, Italy

[3] Department of Clinical and Digital Health Sciences, College of Allied Health Sciences, Augusta University, 987 St. Sebastian Way, EC 4316 Augusta, Georgia 30912

USA

[4]IRCCS Istituto Auxologico Italiano, Department of Geriatrics and Cardiovascular Medicine, via Mosè Bianchi, 90, 20149, Milan Italy

Corresponding Author
s.serino@auxologico.it

1 Supplementary video

After a brief training in VR technology (about 2 minutes), each session comprised two parts, namely an “*encoding phase*” and a “*retrieval phase*”. Starting from the centre of the virtual city, participants were asked to find one, two or three hidden objects (i.e., a bottle of milk, a plant in a vase and a trunk). Participants were specifically instructed to memorize the position of these objects, which were posited at the different parts of the city. Then, in the retrieval phase, they were asked to retrieve the position of the objects identified in first phase (i.e., which were absent) by entering in the virtual city from another starting point.
